# One-step microbial conversion of waste molasses into bioactive fulvic-like acid fertilizer for sustainable soil quality improvement and agricultural waste valorization

**DOI:** 10.3389/fmicb.2026.1998010

**Published:** 2026-10-26

**Authors:** Yuan Zhang, Shijun Pan, Ruinan Wang, Haiming Cheng, Sheng Feng, Huiyan Wang

**Affiliations:** 1 Jilin Collaborative Innovation Center for Antibody Engineering, Jilin Medical University, Jilin, China; 2 Key Laboratory of Straw Comprehensive Utilization and Black Land Conservation, College of Resources and Environmental Science, Education Ministry of China, Jilin Agricultural University, Jilin, China; 3 College of Laboratory, Jilin Medical University, Jilin, China

**Keywords:** biological fermentation, fulvic-like acid, soil carbon metabolism, sustainability, waste molasses

## Abstract

Waste molasses (WM), a carbon rich by-product of sugar production, is frequently underutilized, resulting in environmental pollution and resource loss. In this study, a one-step microbial bioconversion process using *Bacillus subtilis* was developed to transform WM into molasses derived fulvic acid (MFA). The highest MFA yield (33.54%) was achieved at 35 °C, 48 h, and a 5% inoculation rate. Integrated GC-MS and LC-MS analyses revealed dynamic substrate transformation during fermentation and suggested that 4-hydroxy-2-methylacetophenone, 4-oxododecanedioic acid, and 3-hydroxy-2-octylpentanedioic acid were putative intermediates involved in MFA formation. Structural characterization showed that the resulting MFA possessed a loose, porous morphology and abundant hydroxyl, carboxyl, and carbonyl groups. In a single 150 d field trial, MFA application was associated with higher soil organic carbon and humus contents than the unfertilized control and promoted maize vegetative growth. MFA also enriched the microbial communities including *Pseudomonas*, *Acidobacterium*, *Actinobacteria*, and *Bacteroidetes*. The abundance of several genes related to carbon assimilation and carboxylation, including *rbcL*, *rbcS*, *accB*, *accC*, and *pccA*, has also increased. Metabolomic analysis further revealed shifts in metabolites associated with central carbon metabolic pathways, including glycolysis and the pentose phosphate pathway MFA application also markedly promoted maize growth without chemical fertilizer input, demonstrating its potential as an organic soil amendment and partial fertilizer substitute. Collectively, these findings suggest that a one-step microbial transformation can convert WM into functional fulvic-like acid fertilizer. This method provides a potential route for mineral derived FA production. Simultaneously combining the reuse of agricultural waste, improvement of soil quality, reduction of partial fertilizer dependence, and development of circular agriculture.

## 1 Introduction

The continuous expansion of intensive agriculture has substantially increased global food production, but has also accelerated soil degradation, organic matter depletion, and nutrient imbalance, thereby threatening long term agricultural sustainability (Zhang et al., 2025; Albalasmeh et al., 2021; Arshad and Martin, 2002). Improving soil quality while simultaneously reducing the dependence on chemical fertilizers has therefore become one of the major challenges for sustainable agriculture. Among numerous soil amendments, fulvic acid (FA), the most biologically active fraction of humic substances, has attracted increasing attention because of its excellent physicochemical properties and multiple ecological functions (Tiwari et al., 2023). Owing to its relatively low molecular weight and abundant oxygen-containing functional groups, FA can effectively improve soil aggregation, stimulate nutrient availability, enhance microbial activity, and promote plant growth under various environmental conditions (Han et al., 2025; JomehNezhad et al., 2026; Wang et al., 2026). Meng et al. (2025) demonstrated that FA promotes maize seedling growth and improves soil fertility by modulating rare microbial taxa and stimulating bacterial metabolic activity. Jiang et al. (2023) showed that FA can reduce the manganese and copper content in soil and plants, thereby promoting apple plant growth, improving fruit quality and yield. Consequently, FA based fertilizers have been widely recognized as promising biostimulants for improving soil fertility and crop productivity.

Despite these agronomic advantages, the commercial production of FA still relies predominantly on alkaline extraction from non-renewable mineral resources, such as lignite and weathered coal (Li et al., 2026). Li et al. (2024) found that the yields of FA extracted from lignite using potassium hydroxide (KOH) and sodium hydroxide (NaOH) were 26.34 and 24.56%, respectively. Although this extraction technology has been widely adopted, it suffers from several inherent limitations, including high chemical consumption, secondary pollution caused by alkaline wastewater, unstable product quality, and irreversible depletion of fossil carbon resources. These drawbacks have stimulated growing interest in developing renewable and environmentally friendly alternatives for sustainable FA production. Recently, microbial bioconversion has emerged as an attractive strategy because microorganisms are capable of transforming low value organic substrates into humic-like substances through enzymatic oxidation, depolymerization, condensation, and polymerization reactions under mild operating conditions. Compared with conventional chemical extraction, microbial conversion offers advantages including lower energy consumption, fewer chemical inputs, higher environmental compatibility, and greater potential for circular bioeconomy (Gong et al., 2020).

Waste molasses (WM) is an important byproduct of the sugar industry, with a huge output. It is a typical high organic matter byproduct, rich in carbohydrates, organic carbon, and trace elements (Yuan et al., 2026). The world produces a large amount of molasses every year, and the discharge of untreated WM will exacerbate environmental pollution (Shoueir et al., 2022). The existing utilization methods of WM are mostly feed processing, bio refining, and bio chemical preparation. Ren et al. (2025) used WM and inoculated sludge to produce hydrogen gas with an average yield of 348 m^3^d^–1^. Omeroglu et al. (2024) used fermentation technology to produce biofuel 2,3-Butanediol from WM. Xu et al. (2024) used a synthetic enzyme platform to convert WM into glycosides and rare sugars. As a bio refining and biochemical product, there are also problems such as long process chain, high energy consumption, high cost, and insufficient by-product treatment. WM contains a certain amount of amino acids, vitamins, and inorganic ions, which have been proven to be nutrient rich substrates suitable for microbial fermentation (Yan et al., 2011). Previous studies have shown that glucose is hydrolyzed into furfural and 5-hydroxymethylfurfural, which then hydrate to form formic acid, acetic acid, and other small molecule acids, thereby forming precursors for FA synthesis (Yang et al., 2019). The direct microbial transformation of WM into value added humus may provide a more effective and economically attractive utilization pathway.

Previous studies have demonstrated that microbial metabolism can transform carbohydrates into various low molecular weight organic acids, phenolic compounds, and reactive intermediates, which may subsequently undergo oxidative coupling and molecular condensation to generate humic like substances (Hu et al., 2022). Although microbial and hydrothermal approaches have been explored for producing humic and fulvic like substances from agricultural and industrial wastes. Most previous studies have focused on lignocellulosic solid residues and have relied on hydrothermal pretreatment, composting, or multistep physicochemical biological processes. The direct microbial conversion of WM into products rich in FA, as well as the systematic tracking of substrate conversion and subsequent field scale evaluation of soil ecological responses, have not been fully explored. In this study, a one-step microbial bioconversion strategy using *Bacillus subtilis* was established to convert WM into MFA. Fermentation conditions were optimized to maximize the conversion efficiency, while GC-MS, LC-MS and multiple spectroscopic techniques were employed to elucidate substrate transformation and structural evolution during MFA formation. Furthermore, field experiments integrating soil physicochemical analyses, microbial community profiling, functional gene quantification, and metabolomics were conducted to evaluate the effects of MFA on soil quality and carbon metabolism. This work provides mechanistic insights into microbial valorization of WM and demonstrates a sustainable strategy for simultaneously achieving agricultural waste recycling, bioactive fertilizer production, and soil quality improvement.

## 2 Materials and methods

### 2.1 Materials

The molasses liquid comes from the remaining sugarcane molasses after sugar production at Guangxi Sugar Factory in China. The carbon content is 320 g/kg and the nitrogen content is 16.4 g/kg. The composition of WM can be found in Supplementary Material S1. The strains of *Bacillus subtilis* (CICC 10148, China) used for fermentation were purchased from China Center of Industrial Culture Collection. This strain can ferment sugars such as glucose, xylose, and lactose. It also has characteristics such as fermentability and strong protein secretion ability. The cultivation conditions were 30 °C and 48 h, and the initial OD_600_ after 48 h of cultivation is 1.7. Culture medium: protein peptone 5.0 g; beef dipping powder 3.0 g; NaCl 5.0 g; agar 15.0 g; distilled water 1000.0 mL. The culture medium is sterilized at 121 °C for 30 mins, and the WM is sterilized at 115 °C for 15 min. The field experiment was conducted in an experimental field at Jilin Agricultural University in Changchun, China, which had not been fertilized or planted for 5 years. The organic matter content is 11.5 g/kg and the nitrogen content is 0.44 g/kg. The drugs and reagents used in the experiment, such as sodium chloride (NaCl), hydrochloric acid (HCl), sodium hydroxide (NaOH), sodium acetate, acetic acid, potassium sodium tartrate, 3,5-dinitrosalicylic acid, and hydrogen peroxide (H_2_O_2_), are of analytical grade. All purchased from China Sinopharm Chemical Reagent Co., Ltd.

### 2.2 Fermentation experiment

Using the yield of MFA as the response value, three factors were examined: fermentation temperature, fermentation time, and inoculation amount of *Bacillus subtilis*. Fermentation temperature settings: 25 °C, 30 °C, 35 °C, 40 °C and 45 °C. Fermentation time settings: 24 h, 36 h, 48 h, 60 h and 72 h. Inoculation volume settings: 1%, 3%, 5%, 7% and 9% (v/v). On the basis of single factor experiments, Box-Behnken design was used for response surface experiments. Temperature, fermentation time, and inoculum level were selected as the primary optimization variables because they directly influence microbial metabolic activity, substrate conversion duration, and the initial biomass loading, respectively. Agitation and aeration were maintained at constant levels throughout the experiments to ensure consistent aerobic and mixing conditions. Use Design-Expert (13.0, Stat-Ease, USA) software for experimental design, data fitting, and variance analysis to construct a quadratic regression equation. Analyze the impact of various factors and their interactions on the synthesis of MFA, determine the optimal fermentation process parameter combination, and conduct validation experiments. During the cultivation process, the basic conditions were consistent, and all experiments were repeated three times. The fermentation experiment was conducted in a controllable glass fermentation tank with a diameter of 30 cm and a height of 60 cm. The fermentation volume is 25 L, the initial concentration of WM is 20% (v/v), the stirring speed is 200 rpm, the aeration rate is 1.0 VVM, and the pH is controlled at 7.

### 2.3 Field experiment

The experiment was conducted in the experimental field of Jilin Agricultural University, with corn as the experimental crop and a trial period of 150 d. This experimental field has not been fertilized or sown for 5 consecutive years. The experiment adopted a randomized block design with three treatments: a blank control (CK) without any amendments or fertilizers, a group used nitrogen containing compound fertilizers, phosphorus and potassium nutrients of 15-15-15, with a fertilization rate set at 600 kg/hm^2^ (CN), and a 0.2% base application of solid MFA treatment (CF) (Çelik et al., 2008), with three replicates per treatment. The MFA treatment (CF) received MFA as a basal soil amendment at 0.2% (w/w) relative to the dry mass of the 20 cm surface soil layer (Supplementary Material S2). The experimental area is 20 m^2^, and a 50 cm isolation zone is set up between the areas to reduce mutual interference between the processing units. Measure plant growth indicators during the corn harvest period. The individual field plot, rather than individual plants or soil subsamples, was considered the experimental unit. At each sampling time, independently collect soil from each experimental area. Collect data from representative locations within each plot, with a depth of 0–20 cm, avoiding plot boundaries. Homogenize the soil of the same plot to form a composite soil sample. Soil samples for metagenomics and metabolomics analysis were collected at 150 d, with each plot generating an independent composite sample.

### 2.4 Experimental method

The determination of MFA is carried out according to the methods described in agricultural chemical analysis. The quantitative analysis of MFA content is based on the treatment of acid-base solubility and UV visibility (Faithfull, 2003; Jarukas et al., 2021). For detailed methods, please refer to Supplementary Material S3. Use a total organic carbon analyzer (TOC, XPERT-TOC/TNb, TE, Netherlands) to evaluate the carbon content of soil samples and WM. Use a total organic carbon/total nitrogen analyzer to determine the total nitrogen content in WM (Multi N/C 3100, Jena, Germany). Analyze the structural characteristics of MFA using scanning electron microscope (SEM, Regulus8100, Japan), fourier transform infrared spectroscopy (FTIR, Nicolet iS10, Thermo, USA), nuclear magnetic resonance (NMR, EFT-90, Anasazi, USA), and X-ray photoelectron spectroscopy (XPS, K-Alpha, Thermo Scientific, USA). Use an elemental analyzer to analyze the content of C, H, O, and N in MFA (EA, Vario Macro cube, Elementar, Germany). Analyze the components of the fermentation broth using gas chromatography-mass spectrometry (GC-MS, Agilent 7000D, USA) and liquid chromatography-mass spectrometry (LC-MS, TSQ Altis, USA). GC-MS and LC-MS are both qualitative and semi-quantitative analyses.

### 2.5 Microbial analysis

For both metagenomic and metabolomic analyses, three independent field plots per treatment were used as biological replicates (*n* = 3), and one composite soil sample from each plot was collected at 150 d.

Total genomic DNA was extracted from soil samples using the PowerSoil DNA Isolation Kit (Invitrogen, USA) and subjected to shotgun metagenomic sequencing with paired end 150 bp reads. Raw reads were quality filtered using fastp v0.23.1, and potential host-derived reads were removed using Bowtie2 v2.5.4. Clean reads were assembled using MEGAHIT v1.2.9, and open reading frames were predicted from assembled scaftigs using MetaGeneMark. A non-redundant gene catalog was generated using CD-HIT at 95% sequence identity and 90% alignment coverage. Clean reads were mapped back to the gene catalog, and gene abundances were estimated using mapped read counts and gene length with a TPM-like normalization procedure. Taxonomic annotation was performed against the Micro_NR database using DIAMOND and the lowest common ancestor algorithm, whereas functional annotation was conducted against the KEGG, eggNOG, and CAZy databases. Differential taxonomic and functional abundances were analyzed using MetaGenomeSeq. *P*-values were adjusted using the Benjamini-Hochberg false discovery rate procedure, with q < 0.05 considered statistically significant.

Non targeted metabolomics analysis was performed using mass spectrometry (Q ExactiveTM HF/Q ExactiveTM, Thermo, USA) and chromatography (Vanquish UHPLC Thermo, USA). The chromatographic column used is Hypersil GOLD column (100 × 2.1 mm, 1.9 μm). Approximately 1.0 g of soil was extracted with 1.0 ml of 80% methanol, and the supernatant was collected after centrifugation, freeze-dried, and reconstituted in 10% methanol before LC-MS/MS analysis. Pooled quality-control samples were prepared by mixing equal aliquots of all experimental samples, and blank samples were processed in parallel. Raw LC-MS data were converted to mzXML format using ProteoWizard and processed using XCMS for peak detection, quantification, alignment, and retention-time correction. Blank-derived background signals were removed, and relative peak areas were normalized according to total metabolite signal intensity using QC1 as the reference. Features with a CV > 30% among pooled QC samples were excluded. PCA and PLS-DA were performed using metaX. Differential metabolites were defined as those satisfying VIP > 1.0, FC > 1.2 or FC < 0.833, and Benjamini–Hochberg FDR-adjusted *q* < 0.05. KEGG database,^1^ HMDB^2^ and LIPID MAPS database^3^ were used to annotate identified metabolites. Detailed sequencing, LC-MS/MS, database, and computational parameters are provided in Supplementary Material S4.

### 2.6 Statistical analysis

For variables measured repeatedly over time, including SOC and humus content, data were analyzed using a linear mixed effects model with treatment, sampling time, and their interaction as fixed effects. Block was included as a random effect, and plot identity was included as the repeated experimental unit to account for repeated measurements from the same plot. Where significant treatment time interactions were detected, treatment means were compared within each sampling time with appropriate multiple-comparison adjustment.

All statistical analyses were conducted using SPSS data processing software (SPSS 26.0, IBM SPSS, Chicago, USA). Single factor analysis of variance (ANOVA) and Duncan’s multiple range test were used to examine the differences between the treatments, with a significance level set at *p* < 0.05.

## 3 Results

### 3.1 Optimization of MFA production by one-step microbial bioconversion

The effects of fermentation temperature, fermentation time, and *Bacillus subtilis* inoculation rate on MFA production were first evaluated through single-factor experiments. MFA yield increased progressively as the fermentation temperature increased from 25 to 35 °C, reaching a maximum of 31.46%, whereas a further increase to 45 °C resulted in a pronounced decline in yield (Figure 1a). A similar trend was observed for fermentation time (Figure 1b). MFA production increased rapidly during the first 48 h, with the yield rising from 5.17 to 30.26%, followed by a gradual decrease during prolonged fermentation. Likewise, increasing the inoculation rate promoted MFA production up to 5%, while excessive inoculation resulted in reduced yield (Figure 1c; Hu et al., 2024). When the inoculation amount is low, it leads to low biomass and poor fermentation efficiency (Zhang et al., 2021). When the inoculum level is too high, it can easily lead to nutritional competition, insufficient dissolved oxygen (Chen et al., 2022), and inhibit the synthesis of target MFA. These results collectively indicate that MFA formation is highly dependent on balanced microbial metabolic activity rather than continuous biomass accumulation (Skriver et al., 2026; El Salamony et al., 2024). Both insufficient and excessive fermentation conditions negatively affected product formation, suggesting that efficient substrate conversion requires a coordinated balance between microbial growth and extracellular metabolic processes (Keong et al., 2023).

**FIGURE 1:**
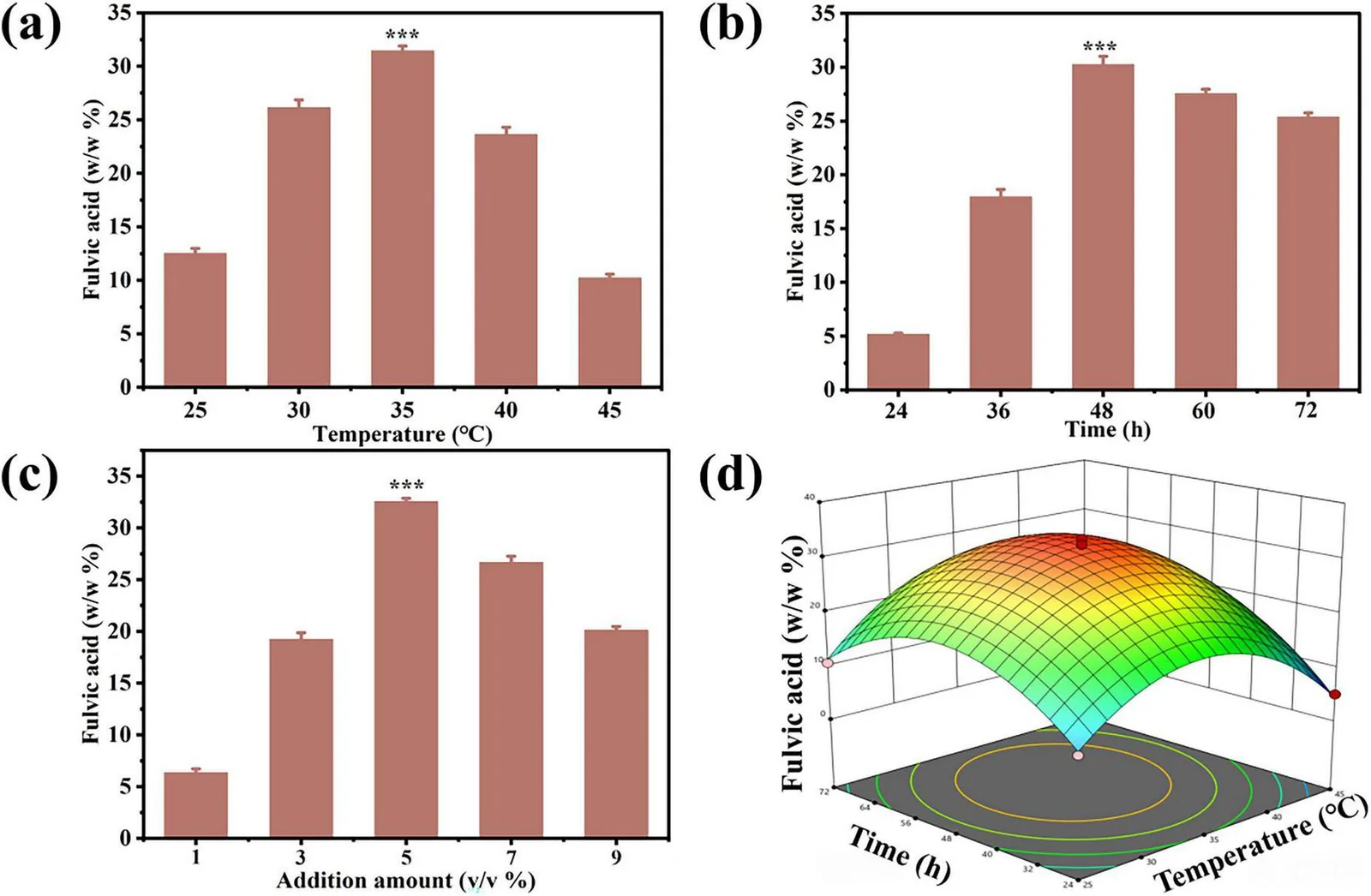
Fermentation experiment of waste molasses with fulvic acid. **(a)** Temperature, **(b)** time, **(c)** addition amount of *Bacillus subtilis*, **(d)** response surface interaction. ***P < 0.001.

To further optimize the fermentation process, response surface methodology (RSM) was employed to evaluate the interactive effects of temperature, fermentation time, and inoculation rate. The elliptical contour plots indicated significant interactions among the three variables (Figure 1d), confirming that MFA production was governed by multiple coupled fermentation parameters rather than any single factor. Under the conditions of 35 °C, 48 h, and 5% inoculation rate, the experimentally determined maximum yield of MFA reached 33.54%, which is very close to the model prediction. For a detailed analysis of Box-Behnken, please refer to Supplementary Material S5.

### 3.2 Enzyme-mediated substrate transformation during WM fermentation

Within 60 h of fermentation of WM, the activity of laccase showed a trend of significant increase followed by a slight decrease (Figure 2a). At 24 h of fermentation, the activity was 40.08 U/L. During this stage, *Bacillus subtilis* was in the logarithmic growth phase, with rapid proliferation as the main mode of bacterial growth. Enzyme synthesis and secretion had just begun (Yang et al., 2025). Fermentation lasts for 48 h, entering a stable growth phase, where secondary metabolism and extracellular enzyme secretion reach their peak, with activity increasing to 106.52 U/L. The carbon source and metabolic intermediates in WM provide sufficient substrate and energy support for laccase synthesis (Bai et al., 2024). At 60 h of fermentation, a large amount of nutrients were consumed in the WM, and the accumulation of metabolic by-products inhibited enzyme production, resulting in a decrease in enzyme activity to 87.64 U/L. The activity of invertase also follows the pattern of first increasing and then decreasing (Figure 2b), with an activity of 3.23 U/mL at 24 h of fermentation. The enzyme synthesis efficiency is relatively low due to the metabolic characteristics of *Bacillus subtilis*, which mainly rely on proliferation during logarithmic growth. At 48 h, it entered a stable period, and the sucrose substrate in the WM induced the strain to secrete a large amount of invertase (Kabir et al., 2025), resulting in an activity of 8.91 U/mL. At 60 h, the substrate was almost depleted and decreased to 5.06 U/mL.

**FIGURE 2:**
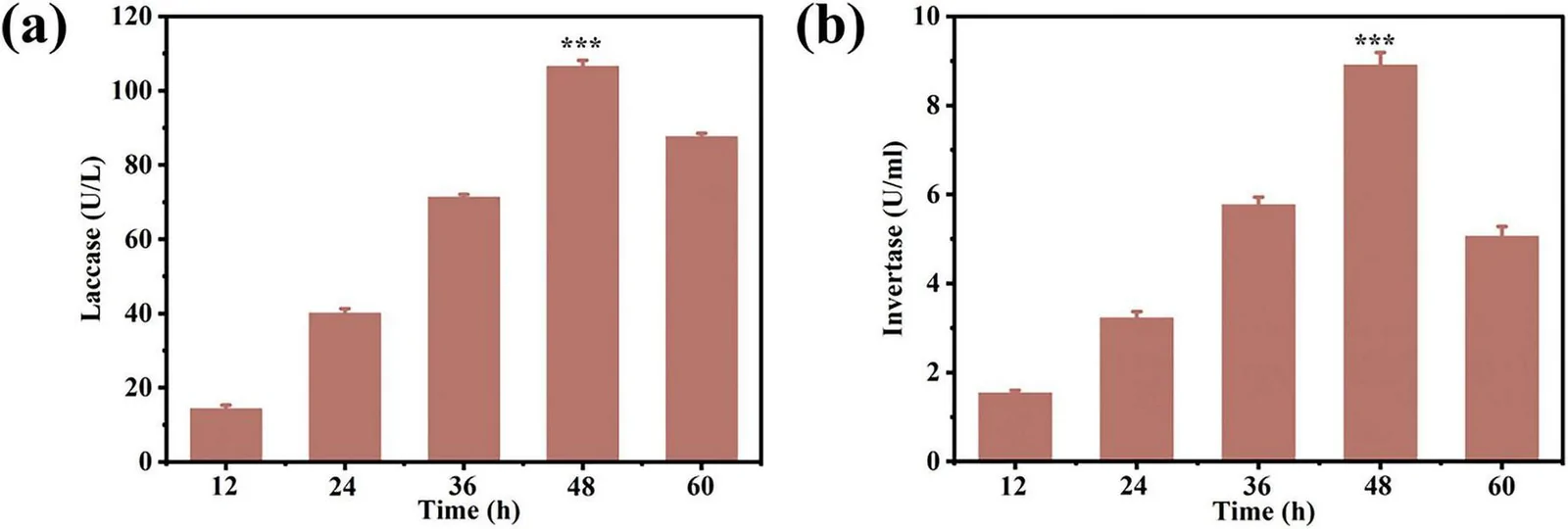
Changes in enzyme activity during the fermentation process of waste molasses: **(a)** laccase, **(b)** invertase. ***P < 0.001.

GC-MS (Supplementary Material S6) and LC-MS (Supplementary Material S7) techniques were used to detect the relative content of major substances in WM before fermentation, after 24 h of fermentation, and after 48 h of fermentation. In GC-MS, butanoic acid, 2-methyl-, ethyl ester continued to decrease from 8.49 to 1.10%, butanoic acid, 2-methyl-, propyl ester continued to decrease from 6.16 to 1.84%, phenol, 2-methoxy continued to decrease from 10.02 to 0.11%, phenol, 4-ethyl continued to decrease from 9.13 to 1.34%, and phenol, 2,6-dimethoxy continued to decrease from 15.48 to 1.05%. These substances are the main volatile substances in WM, and the continuous decrease indicates that they are continuously decomposed under the action of *Bacillus subtilis* (Zhang et al., 2012; Wu et al., 2017; Kwon et al., 2025; Guo et al., 2022). Tetradecane is a metabolite produced by the continuous decomposition of long-chain fatty acids in WM by *Bacillus subtilis*, which has not been utilized throughout the process. Its content has increased from 2.66 to 11.93%. Decanoic acid, ethyl ester increased from 2.58 to 4.26% and then decreased to 0.53%, 1-dodecanol increased from 6.54 to 8.22% and then decreased to 3.69%, 1-tetradecanol increased from 9.11 to 9.41% and then decreased to 1.22%, heptadecane increased from 2.57 to 4.32% and then decreased to 0.49%, hexanedioic acid, bis (2-methylpropyl) ester increased from 1.07 to 6.54% and then decreased to 0.86%, 4-Hydroxy-2-methylacetophenone increased from 8.55 to 41.57% and then decreased to 4.37%, nonanal increased from 2.77 to 10.09% and then decreased to 0.26%, and dodecanal increased from 3.20 to 5.31% and then decreased to 0.17%. The trends of these substances all show an initial increase followed by a decrease. This indicates that they are all products produced by the decomposition of WM by *Bacillus subtilis*, and are precursor substances directly involved in the synthesis of MFA during the transformation process (Pan et al., 2025; Zhao et al., 2022; Cao et al., 2026).

In LC-MS, sinapinic acid decreased from 18.36 to 1.25%, syringic acid decreased from 12.77 to 0.38%, 2,6-dimethoxyphenol decreased from 12.94 to 1.06%, ethyl vanillate decreased from 13.24 to 3.05%, L-α-palmitin decreased from 6.52 to 2.04%, L-phenylalanine decreased from 6.39 to 3.07%, L-isoleucine decreased from 2.66 to 0.47%, and L-tyrosine decreased from 4.24 to 1.02%. These substances are the main organic acids, phenols, lipids, and amino acids in WM (Liu et al., 2025; Stoilova et al., 2006). The overall trend is declining, providing a favorable living environment for *Bacillus subtilis*. 4-oxododecanedioic acid increased from 1.59 to 28.32% and then decreased to 10.26%, 3-hydroxy-2-octylpentanedioic acid increased from 3.23 to 25.84% and then decreased to 7.35%, and 3-(4-hydroxy-3-methoxyphenyl) propanoic acid increased from 0.12 to 8.61% and then decreased to 2.73%. These substances are the decomposition products of lipids and carboxylic acids in WM, which directly participate in the synthesis of MFA.

Combining the results of GC-MS and LC-MS, phenol-2-methoxy, phenol-2,6-dimethoxy, sinapinic acid, syringic acid, 2,6-dimethoxyphenol, Ethyl- vanilla is the main substance in WM. These substances are the core raw materials for fermentation and are continuously degraded by *Bacillus subtilis*, providing carbon, nitrogen, and energy sources for subsequent metabolism. The transient accumulation and subsequent decline of 4-hydroxy-2-methylacetophenone, 4-oxododecanedioic acid, and 3-hydroxy-2-octylpentanedioic acid suggest that these compounds may represent putative intermediates associated with the transformation of waste molasses during MFA formation. These substances ultimately undergo biochemical reactions such as oxidation, condensation, and polymerization to form MFA containing active functional groups such as carboxyl, hydroxyl, and methoxy groups.

### 3.3 MFA structural features

Observation of the microstructure of MFA prepared by fermentation of WM. The SEM image (Figure 3a) shows that MFA exhibits a loose and porous morphology. This porous structure is formed by the metabolic process and substance transformation of microorganisms during fermentation, which not only provides a large specific surface area for MFA, but also gives it good adsorption performance and reaction activity. In Fourier transform infrared spectroscopy (Figure 3b), the vibration at 3500–3400 cm^–1^ is attributed to the hydroxyl (−OH) groups converted from WM (Tucureanu et al., 2016). The functional groups of carbonyl (C = O) and carboxyl (−COOH) cause vibrations at 1600–1500 cm^–1^. And during the self oxidation process, phenolic hydroxyl groups are oxidized to quinone groups, resulting in spectral absorption in the 1500–1300 cm^–1^ region of the conjugated system (Lievens et al., 2011). The vibration in the 1100–1000 cm^–1^ region is mainly due to the synergistic contribution of aromatic ether bonds and aliphatic C-O stretching vibrations. This is the core spectral marker of the evolution of MFA molecules from simple phenolic acids to complex aromatic ether network structures (Brebu et al., 2025). The observed characteristic absorption peaks of –OH, C = O and –COOH confirm the occurrence of oxidation and condensation reactions during the fermentation process of WM. *Bacillus subtilis* converts sugars and nitrogen-containing compounds in raw materials into oxygen-containing functional groups and aromatic structures unique to MFA, which are the structural basis for the acidic and chelating properties of MFA. NMR is considered a useful tool for evaluating the structural properties of organic substances (Figure 3c). It can usually be divided into four regions, namely alkyl carbon (0–50 ppm), oxidized aliphatic carbon (50–100 ppm), aromatic carbon (100–160 ppm) and carboxyl/carbonyl carbon (160–220 ppm) (Chai et al., 2008). The spectrum of MFA shows strong vibrations of alkyl carbon and carbonyl carbon, weak vibrations of adjacent alkyl carbon, and no characteristic signals of methoxy carbon and aromatic carbon, depending on the raw material composition and fermentation conversion mechanism of WM. The main components of WM are soluble sugars such as sucrose, glucose and fructose. During the fermentation process, microorganisms convert the sugar substances in molasses into intermediate products such as fatty organic acids, alcohols, esters, etc. These intermediate products further polymerize to form long-chain alkyl structures, significantly enhancing the resonance vibration signal of alkyl carbon. Meanwhile, the fermentation process of WM is an aerobic oxidation process, and the metabolic action of microorganisms can efficiently catalyze the oxidation reaction of intermediate products, generating a large number of carbonyl functional groups such as carboxyl and quinone groups, exhibiting strong resonance vibration signals. WM contains almost no traditional aromatic carbon precursors such as lignin, polyphenols, and aromatic compounds, and does not have the material basis to generate a large amount of aromatic carbon structures. And *Bacillus subtilis* does not possess the key enzyme system for lignin degradation and aromatic ring reconstruction, and has not undergone dehydrogenation, cyclization, and aromatization reactions of aromatic rings, thus unable to produce aromatic carbon signals.

**FIGURE 3:**
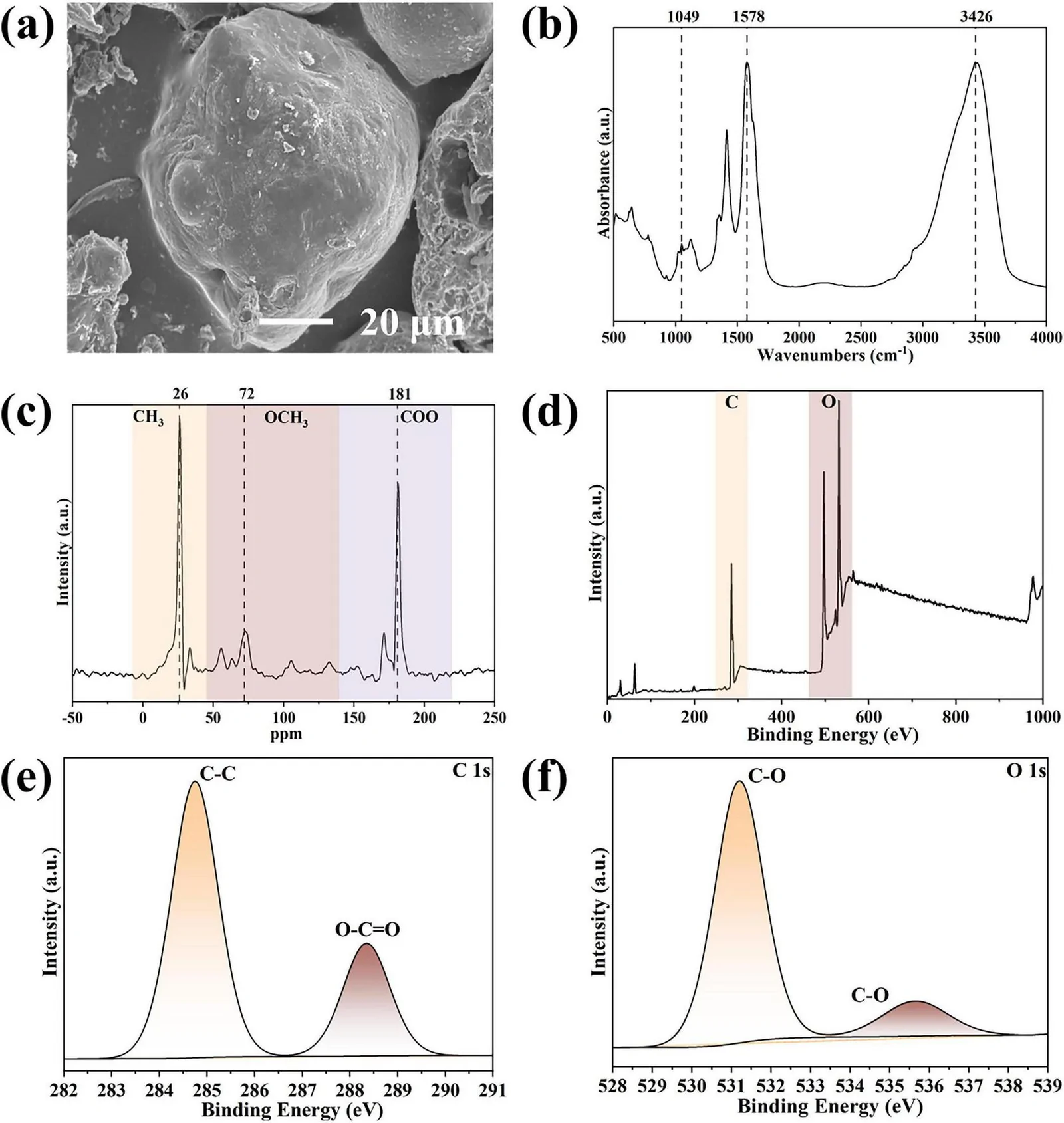
Structural characteristics of fulvic acid prepared from waste molasses: **(a)** scanning electron microscope, **(b)** fourier transform infrared spectroscopy, **(c)** ^13^C nuclear magnetic resonance, **(d)** X-ray photoelectron spectroscopy survey scan, **(e)** X-ray photoelectron spectroscopy C 1s, **(f)** X-ray photoelectron spectroscopy O 1s scan.

In the XPS spectrum of MFA, the two peaks located at 285 eV and 532 eV correspond to C1s and O1s, respectively (Figure 3d). The C 1s peak after deconvolution can be decomposed into peaks of 284.5 eV, 286.2 eV, and 288 eV, which are C-C and C-O, respectively (Figure 3e). The O 1s peak can be decomposed into peaks of 531.2 eV and 535.5 eV (Figure 3f). In biological fermentation, *Bacillus subtilis* can metabolize and decompose organic matter, and it is easier to utilize and degrade oxygen-containing functional groups. More organic compounds with relatively simple structures are used as carbon sources. The structurally stable and hydrophobic C-C is more difficult to degrade by microorganisms, while the C-C structure is relatively enriched (Li et al., 2021). During this process, small organic molecules will further aggregate and condense under the action of microorganisms, forming more complex and stable humus macromolecules. Various enzymes secreted by microorganisms catalyze the further oxidation of residual alcohol hydroxyl or aldehyde groups (-CHO) in hydrothermal products, producing –COOH or ketone groups (C = O). This biological oxidation process increases the relative content of surface O-C = O functional groups (Shutthanandan et al., 2019). As a byproduct of the sugar industry, WM is mainly composed of soluble sugars such as sucrose, glucose and fructose. Its molecular structure contains a large amount of –OH, providing sufficient precursors for the formation of O-C = O (Zhu et al., 2017). The fermentation process is an aerobic oxidation process, and the metabolic action of microorganisms can efficiently catalyze the oxidation reaction of sugar substances. –OH is oxidized to aldehyde groups, further oxidized to carboxyl groups, and some carboxyl groups undergo esterification reactions with alcohol substances generated during fermentation, forming ester groups. The O-C = O bond in the carboxyl and ester groups together constitute the main source of this characteristic peak in XPS spectra. WM is mainly composed of sugar substances, and the oxygen-containing functional groups generated during fermentation and transformation are mainly C = O and –COOH, so the amount of C-O bond formation is limited.

Elemental analysis (Supplementary Material S8) showed that the isolated MFA contained 32.61% C, 4.40% H, 30.58% O, and 1.21% N. The relatively high oxygen content was consistent with the abundance of oxygen-containing functional groups identified by FTIR, NMR, and XPS analyses. The calculated atomic H/C and O/C ratios were approximately 1.61 and 0.70, respectively, while the C/N ratio was approximately 31.4. The relatively high O/C ratio indicates a high degree of oxygenation and is consistent with the presence of abundant carboxyl, hydroxyl, and carbonyl groups. Meanwhile, the H/C ratio suggests that the MFA possessed a predominantly aliphatic character with contributions from unsaturated and aromatic structures. The low nitrogen content further indicates that nitrogen containing components represented only a minor fraction of the isolated product.

### 3.4 Effects of MFA on soil organic carbon and humus accumulation

By setting up a blank control group (CK) without fertilization, a compound fertilizer treatment group (CN), and a MFA treatment group (CF), the effect of MFA on soil quality was explored. Soil organic carbon (SOC) is commonly used to assess soil quality (Figure 4a; Zhang et al., 2026a). The CK group remained at the baseline level, reaching 12.81 g/kg at 30 d, continuously decreasing to 10.5 g/kg at 120 d, and recovering to 11.24 g/kg at 150 d. Due to the absence of fertilization and planting for 5 years, there is no external input of organic carbon into the soil, and the original organic carbon is in a state of slight consumption under the action of microbial decomposition, resulting in a low and stable overall content. The SOC content of CN group was 15.24 g/kg at 30 d, continuously decreased to 12.39 g/kg at 120 d, and rebounded to 14.93 g/kg at 150 d. Compared with the CK group, it increased by 32.83%. Although the application of chemical fertilizers is mainly based on inorganic nutrients, it can promote microbial activity by supplementing nutrients from external sources (Zhang et al., 2026b). Thus, to a certain extent, the mineralization loss of SOC can be reduced, and the SOC content can be maintained and increased. The SOC content in the CF group was 17.55 g/kg at 30 d, continuously decreased to 13.76 g/kg at 120 d, and rebounded to 15.27 g/kg at 150 d. Compared with the CK group, it increased by 35.85%, and compared with the CN group, it increased by 2.28%. MFA itself belongs to organic carbon substances, and after application, it directly supplements external organic carbon to the soil (Liu et al., 2026). Meanwhile, MFA can promote soil microbial activity, enhance soil ability to retain organic carbon, and significantly increase SOC storage.

**FIGURE 4:**
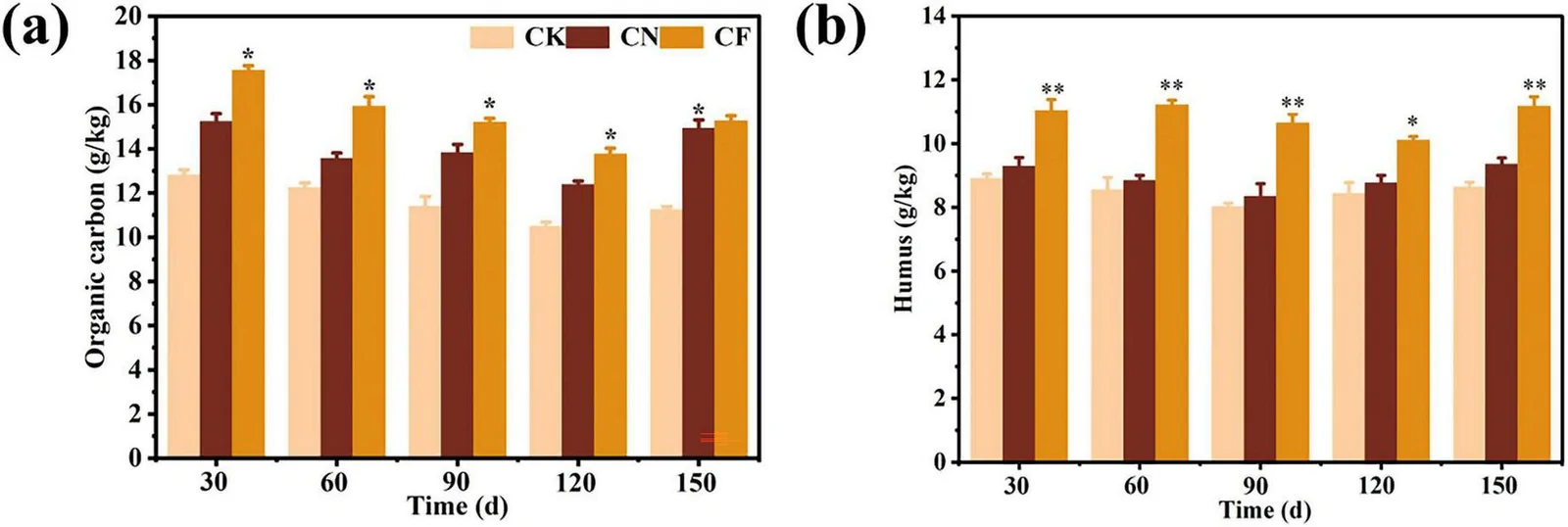
Changes in soil quality of CK, CN and CF: **(a)** soil organic carbon, **(b)** soil humus. Data are presented as mean ± SD of three independent biological replicates (*n* = 3). Statistical differences among treatments at each sampling time were evaluated by one-way ANOVA followed by Duncan’s multiple range test. Asterisks indicate significant differences relative to CK at the same sampling time (*P < 0.05; *P* < 0.01).

From the analysis of changes in soil humus (HS) content (Figure 4b), the CK group had the lowest HS content of 8.64 g/kg at 150 d. When there is no external input of substances, there is insufficient raw material for the synthesis of HS in the soil, making it difficult for microorganisms to form new HS through decomposition and transformation, resulting in a low content. At 150 d, the HS content in the CN group was 9.35 g/kg, a slight increase of 8.22% compared to the CK group. The application of compound fertilizer provided inorganic nutrients for the growth and reproduction of soil microorganisms, enhanced their ability to decompose and transform small amounts of organic matter in the soil, and promoted the synthesis of small amounts of HS, resulting in a slight increase in HS content (He et al., 2025). However, due to the lack of direct organic substrates, the improvement effect is limited. At 150 d, the HS content in the CF group was 11.17 g/kg, an increase of 29.28% compared to the CK group and 19.47% compared to the CN group. MFA is an important component of HS, and direct application can rapidly increase the total amount of soil HS. At the same time, MFA, as an active organic substance, can provide sufficient carbon and energy sources for microorganisms involved in HS synthesis, accelerate the humification process of organic matter in soil, and promote the continuous synthesis of HS (Meng et al., 2026).

### 3.5 Recombination and metabolic reactions of soil microbial communities

The soil microbial communities of CK, CN, and CF showed distinct clustering patterns in the PCA ordination (Figure 5a), with samples from the same treatment tending to cluster together and samples from different treatments showing varying degrees of separation. Compared to the CK and CN, the CF significantly upregulated the relative abundance of several beneficial soil functional bacteria, including *Pseudomonas*, *Acidobacteriota*, *Actinomycota* and *Bacteroidota* (Figure 5b). According to reports, certain diazotrophic strains of *Pseudomonas* can help plants better utilize the existing nitrogen in the soil, thereby promoting plant growth (Adrien et al., 2025). Some *Pseudomonas* strains can also interact closely with plant roots and participate in plant-associated nitrogen cycling, which may contribute to plant growth and nutrient turnover (Dong et al., 2026). *Acidobacteriota* can degrade polysaccharides in plant residues such as cellulose, promote the decomposition of humus, and promote soil carbon cycling (Gonçalves et al., 2024). *Actinomycota* can secrete various extracellular enzymes, promote the decomposition and transformation of soil organic matter, and release available nutrients for plants and other microorganisms to utilize (Ji et al., 2026). At the same time, it can produce antibacterial substances, inhibit the reproduction of pathogenic microorganisms in the soil, and enhance the stability of soil microecology (Wang et al., 2025). *Bacteroidota* can efficiently degrade complex organic compounds such as polysaccharides and humic acids in soil, participate in the formation and transformation of soil HS, improve soil particle structure, and enhance soil fertility and water retention capacity (Kruczyńska et al., 2023). MFA not only provides nutrients for microorganisms, but also improves soil pH, aeration and other microbial environments, making it more suitable for the colonization, reproduction and metabolism of various functional bacteria. However, the CN only supplements inorganic nutrients such as nitrogen, phosphorus, and potassium through fertilizers, and cannot provide organic carbon sources for microorganisms. Moreover, a single fertilizer input can easily lead to soil microbial imbalance, only slightly increasing the abundance of some tolerant microorganisms. The relative abundances of several genes associated with carbon assimilation and carboxylation, including *rbcL*, *rbcS*, *accB*, *accC*, and *pccA*, were higher in the CF treatment than in CK and CN (Figure 5c; Wang et al., 2023).

**FIGURE 5:**
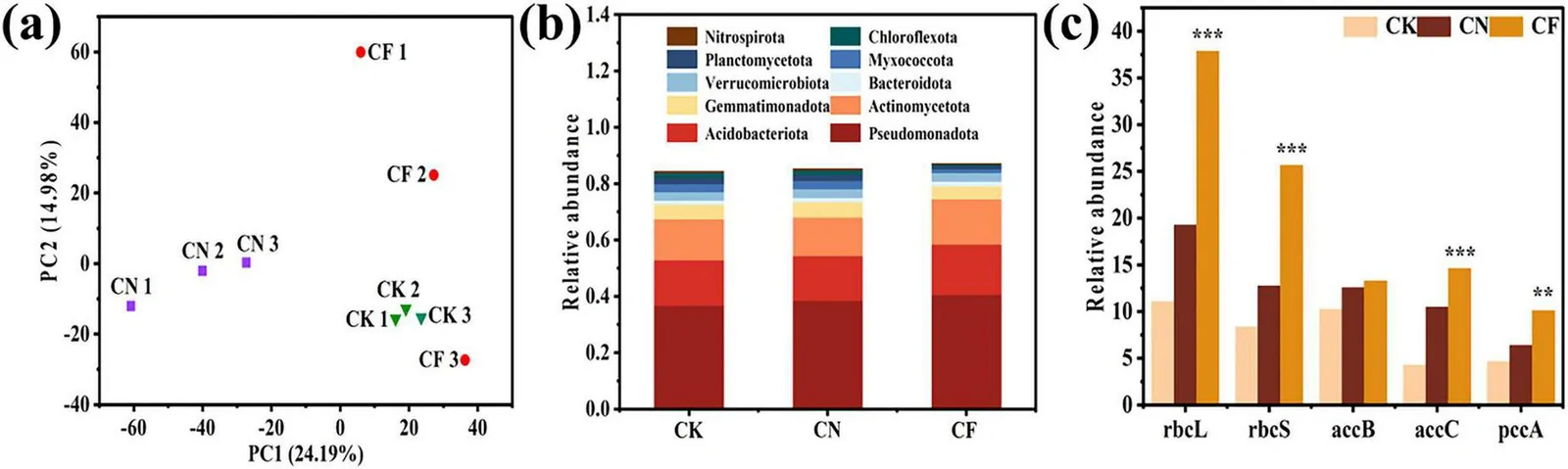
Macro genomic analysis of soil microorganisms: **(a)** PCA principal component analysis, **(b)** microbial phylum level richness, **(c)** relative abundance of related genes based on metagenomic sequencing. **P < 0.01, ***P < 0.001.

CK, CN and CF show a significant separation trend in principal component space, with tightly clustered intra group repeats and high inter group discrimination (Figure 6a). Different treatments can significantly alter the metabolic profile characteristics of soil as a whole, and there are significant differences in metabolism between CF treatment and CK and CN. The addition of MFA can induce unique metabolic responses in plants, which is different from the conventional mode of no fertilization and single application of chemical fertilizers. In terms of metabolites (Figure 6b), compared to the CK and CN groups, CF significantly upregulated D-(+)-Galactosamine, 2- (4-hydroxyphenyl)-1-nitroethane, vidarabine, arbutin. These substances are all related to the synthesis and accumulation of key metabolites such as amino acids, organic acids and sugars. In KEGG analysis (Figure 6c), CF significantly Improved multiple metabolic pathways. Mainly focused on core pathways such as carbon metabolism, nitrogen metabolism, energy metabolism and secondary metabolism biosynthesis. In the most important metabolic pathways (Figure 6d), MFA significantly enhances Salicin, Glycerone-P and D-Ribose in the Glycolysis and Pentose phosphate pathways. This is related to the fixed CO_2_ and Calvin cycle operation. Metabolomic analysis showed altered abundances of metabolites associated with glycolysis, the pentose phosphate pathway, and other central carbon metabolic pathways. These changes suggest that MFA treatment was associated with shifts in soil microbial carbon metabolism.

**FIGURE 6:**
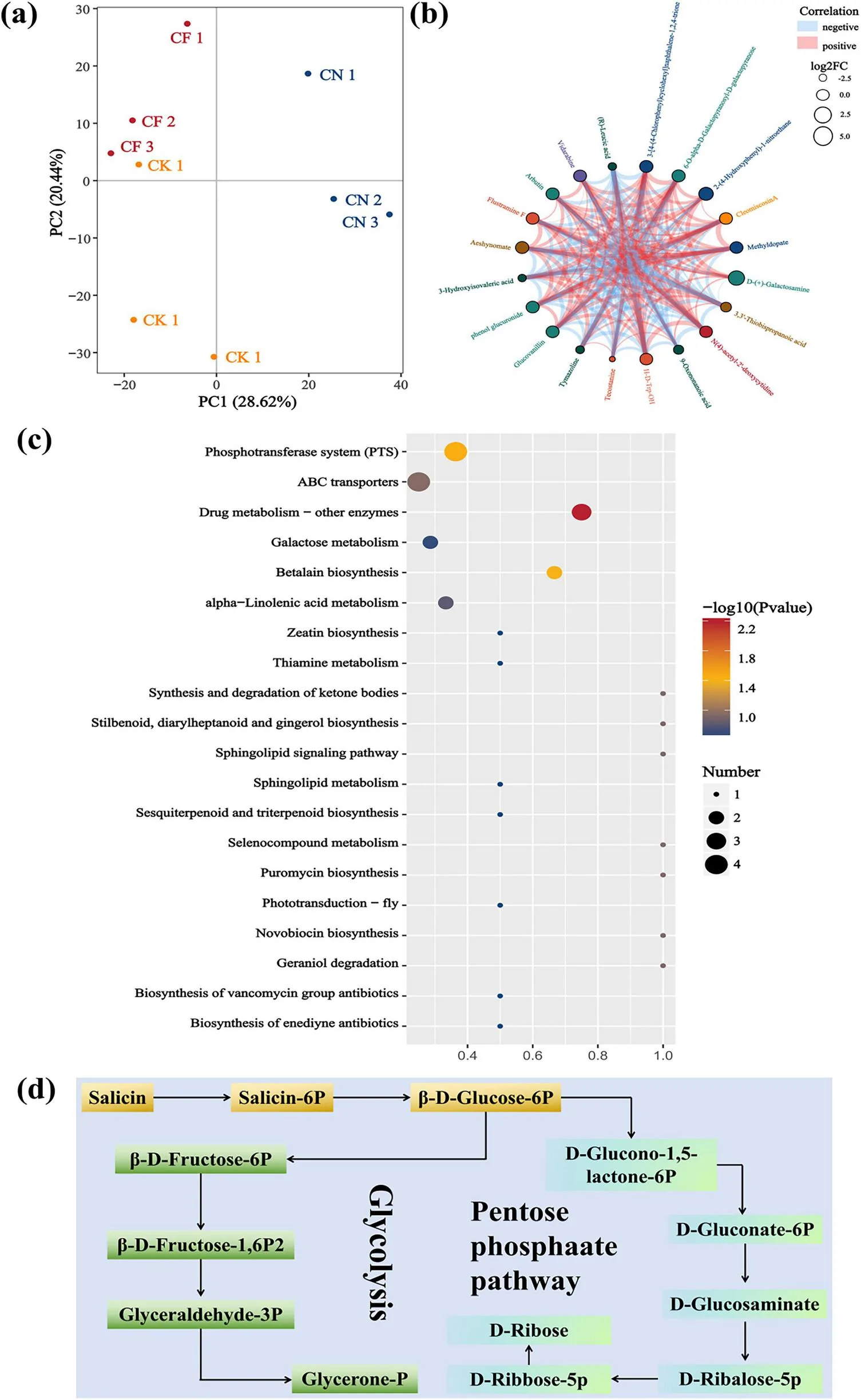
Soil microbial metabolomics: **(a)** PCA principal component analysis, **(b)** CF upregulates metabolites, **(c)** CF upregulates KEGG pathway, **(d)** metabolic pathways associated with soil carbon metabolism in the CF treatment.

### 3.6 Plant growth status

Different treatments have significant effects on the plant height, stem thickness, ear position, leaf length, and aboveground dry matter mass of corn during its 150 d growth period (Supplementary Material S9). The plant height, stem thickness, leaf length and dry matter mass of CK are 142.34 cm, 1.16 cm, 50.93 cm and 110.52 g. Due to the continuous depletion of soil nutrients, plant growth is severely inhibited, and all growth indicators are the lowest. The plant height, stem thickness, leaf length and dry matter mass of CN are 248.51 cm, 2.63 cm, 88.36 cm and 284.26 g. Compound fertilizer can quickly supplement soil available nutrients and significantly improve the growth status of corn. The plant height, stem thickness, leaf length and dry matter mass of CF are 230.06 cm, 2.41 cm, 80.18 cm, and 237.96 g, respectively. The growth of corn is significantly better than that of CK, although slightly lower than that of CN, the overall difference is small and the performance is similar, without obvious growth weakness. MFA can effectively improve the structure of barren soil, enhance soil quality and nutrient supply capacity. Without the use of fertilizers, it can still significantly promote the growth and development of corn, and may reduce dependence on fertilizers, achieving increased crop yield and improved quality.

## 4 Discussion

The core mechanism of one-step biotransformation of WM to FA lies in the enzymatic catalysis and substrate directed reconstruction of *Bacillus subtilis*. WM provides a chemically complex substrate dominated by readily fermentable carbohydrates but also containing phenolic compounds, organic acids, amino acids, lipids, and inorganic nutrients. The inoculum level showed a clear nonlinear effect on MFA production. At relatively low inoculum levels, the limited initial biomass may have delayed microbial proliferation, extracellular enzyme accumulation, and substrate conversion, resulting in insufficient transformation of the waste molasses within the fermentation period. Increasing the inoculum to 5% (v/v) likely provided sufficient active biomass to rapidly initiate substrate utilization and MFA forming reactions. However, further increasing the inoculum may have increased competition for available carbon and nitrogen sources and raised the oxygen demand of the culture. Because agitation and aeration were maintained at fixed levels, excessive biomass could also have increased the risk of oxygen-transfer limitation. These combined effects may explain the decline in MFA conversion observed at inoculum levels above 5%. Thus, under the present operating conditions, a 5% inoculum likely represented an appropriate balance among initial biomass, nutrient availability, and oxygen supply. During fermentation, these components were not transformed uniformly. Instead, the simultaneous depletion of native molasses constituents and transient accumulation of several low molecular weight compounds indicates a dynamic redistribution of carbon through multiple metabolic routes. The temporal accumulation patterns of several acetophenone- and dicarboxylic acid related compounds were consistent with transient intermediate formation during waste-molasses fermentation. They are more appropriately considered candidate intermediates within the broader network of microbial oxidation, degradation, and condensation reactions occurring during fermentation. Hydrolytic and oxidative reactions jointly controlled this transformation. Invertase likely increased the accessibility of sucrose derived carbon by producing fermentable monosaccharides, thereby supporting microbial growth and the generation of primary metabolites (Kabir et al., 2025). These products may subsequently have been redirected toward organic acids, aldehydes, ketones, alcohols, and lipid derived compounds. In parallel, the increase in laccase activity suggests that oxidative reactions occurred during the same stage in which MFA accumulated. Laccase mediated oxidation may have converted susceptible phenolic compounds into phenoxy radicals or quinone like structures, which are chemically more reactive and can participate in coupling, nucleophilic addition, esterification, and condensation reactions (Jeon et al., 2012). The enrichment of hydroxyl, carboxyl, carbonyl, and C-O containing groups indicates that the conversion process increased the chemical functionality of the original carbon substrate. However, the weak aromatic carbon signal detected by NMR argues against extensive aromatization or formation of highly condensed aromatic macromolecules. Instead, the product is more reasonably interpreted as an oxygen-rich, predominantly aliphatic FA like assemblage derived from carbohydrate fermentation and secondary oxidation. Its agronomic activity may therefore arise primarily from high oxygen functionality, molecular heterogeneity, and chemical reactivity rather than aromatic condensation.

From a contemporary humic substance perspective, MFA should not necessarily be considered a single covalently linked macromolecule. A more plausible model is a supramolecular association of low and intermediate molecular weight compounds stabilized by hydrogen bonding, hydrophobic interactions, metal bridging, ester linkages, and partial oxidative coupling (Sutton and Sposito, 2005). In this model, microbial fermentation does not simply synthesize MFA. Instead, it restructures the original molasses carbon into a more chemically heterogeneous and functionally reactive organic fraction. After soil application, MFA likely influenced soil carbon dynamics through two parallel mechanisms, direct organic carbon addition and indirect biological transformation. The chemical heterogeneity of MFA may be central to this dual behavior. Its relatively labile fractions can serve as readily available carbon and energy sources, stimulating microbial growth and substrate turnover. Simultaneously, its carboxyl-, hydroxyl-, and carbonyl rich fractions may interact with clay minerals, metal ions, and existing organic matter through ligand exchange, cation bridging, hydrogen bonding, and surface complexation (Kleber et al., 2021). These interactions can alter the accessibility of organic substrates and potentially increase their residence time in soil. Thus, MFA may simultaneously promote short term microbial metabolism and contribute to longer term carbon retention, depending on the balance between mineralization, microbial assimilation, residue formation, and mineral association. This balance is likely mediated by microbial carbon use efficiency (He et al., 2024). When labile MFA derived carbon is metabolized, part of it is released as CO_2_, whereas another part is incorporated into microbial biomass and metabolites. Upon microbial turnover, cellular residues and necromass can interact with mineral surfaces and become important precursors of persistent soil organic matter.

Although the MFA yield reached 33.54% under the optimized conditions, the fraction not recovered as MFA should not simply be regarded as unconverted waste. This residual fraction may include unconsumed sugars and other soluble organic compounds, microbial biomass, low molecular weight metabolites, other non MFA organic fractions. At the primary fermentation stage, further improvement in MFA production could be achieved by optimizing substrate loading, feeding strategy, nutrient balance, agitation, aeration, and dissolved oxygen supply. In particular, fed batch operation may provide a means of maintaining substrate concentrations within a favorable range while avoiding excessive osmotic stress, nutrient imbalance, and oxygen demand associated with high initial molasses loading. In addition to maximizing the formation of MFA, we also envisioned a cascade stabilization strategy for the remaining fermentation components. Residual fermentable sugars and low molecular weight organic compounds can undergo secondary biotransformation steps to produce additional value added metabolites or multi-level energy carriers (Aghajanyan et al., 2026). The residual organic matter from fermentation can be combined with anaerobic digestion, composting, or other humification processes to produce soil improvement products (Malik et al., 2019). This cascading method will transform the process from single product MFA fermentation to a comprehensive waste molasses biorefinery.

From a biorefinery perspective, the major significance of this work lies in coupling waste valorization with soil functionality. Instead of treating molasses solely as a fermentation feedstock for a purified chemical product, the process converts a heterogeneous industrial by-product into a functionally complex amendment whose value derives from both its chemical properties and its ability to reshape soil biological processes. The mechanistic value of this study therefore lies not only in producing a FA fertilizer, but in demonstrating how microbial conversion can transform an industrial sugar by-product into a biologically active material capable of coupling waste carbon reutilization with soil ecological restoration. Nevertheless, this study also has some limitations. Firstly, no comparative strain screening experiments were conducted. Therefore, current research results have confirmed the feasibility of converting waste molasses, but have not proven it to be the optimal strain for producing MFA. Future research should systematically screen multiple *Bacillus* strains and other candidate microorganisms based on sugar utilization efficiency, MFA production, extracellular enzyme production, substrate tolerance, biomass formation, and oxygen demand. Secondly, process optimization is limited to fermentation temperature, fermentation time, and inoculation amount. Mixing, aeration, dissolved oxygen, oxygen transfer capacity, substrate loading strategy, and nutrient balance are maintained as fixed or unmeasured variables, and therefore remain important goals for further optimization. Future bioreactor research should combine DO monitoring, OTR, OUR, and kLa measurement with batch and feed feeding strategies to improve substrate utilization and process scalability. Thirdly, a complete carbon mass balance was not established in the present study. Carbon from waste molasses that was not recovered in the MFA fraction may have remained in residual soluble substrates, microbial biomass, low molecular weight metabolites, or other organic products, or may have been released as CO_2_. In addition, although several candidate intermediates associated with MFA formation were identified, direct precursor product relationships were not demonstrated. Future studies should therefore combine stable isotope labeling with metabolic flux analysis to trace carbon partitioning among MFA, microbial biomass, residual substrates, metabolites, and CO_2_, and to determine whether the proposed intermediates contribute directly to MFA formation. Improved fractionation and purification procedures should also be incorporated to better define the chemical composition of the recovered MFA fraction. Fourthly, strain improvement is an important opportunity to increase MFA production. Adaptive laboratory evolution, classical mutagenesis, and metabolic or synthetic biology methods can be used to enhance tolerance to high osmotic sugar syrup environments, improve carbohydrate absorption and utilization, enhance oxidative and extracellular enzyme capabilities, and redirect intracellular carbon allocation to MFA related transformation pathways. Fifth, residual salts, unused sugars, low molecular weight organic acids, and other acid soluble compounds may contribute to the recovered dry matter, and their respective contributions were not quantified in this study. Future research will combine desalination, dialysis with chromatography or molecular weight based fractionation to improve the chemical definition and purity evaluation of recovered MFA like fractions. Finally, the field evaluation was limited to a single 150 d maize growing season, and the degradation dynamics, persistence, nutrient release behavior, and agronomic effects of MFA under different climatic and soil conditions were not examined. The cumulative effects of long-term MFA application on soil physicochemical properties and microbial communities therefore remain unclear. Future studies should extend the duration of field trials and conduct multisite, multiyear experiments across contrasting climate zones, soil types, and cropping systems. Such studies will be necessary to determine the long term effects of MFA on soil properties, microbial community structure, and ecological functions, and to establish application strategies appropriate for different agricultural environments.

## 5 Conclusion

This study successfully prepared FA from WM using a one-step fermentation method with *Bacillus subtilis*, and achieved practical application. Under the optimal conditions of fermentation temperature of 35 °C, time of 48 h, and inoculation volume of 5%, the MFA yield reached 33.54%. 4-Hydroxy-2-methylacetophenone, 4-oxododecanedioic acid and 3-hydroxy-2-octylpentanedioic acid are putative intermediates associated with MFA formation. The prepared MFA exhibited a loose and porous microstructure and abundant oxygen containing functional groups. In the single 150 d field trial, MFA treatment was associated with a 35.85% higher SOC content and a 29.28% higher humus content than the unfertilized control at 150 d, together with improved maize vegetative growth. And it is related to changes in the composition of soil microbial communities, including increased relative abundances of *Pseudomonas*, *Acidobacteriota*, *Actinomycetota*, and *Bacteroidota*. MFA treatment was also associated with higher abundances of *rbcL*, *rbcS*, *accB*, *accC*, and *pccA*, indicating an enrichment of genetic potential related to carbon assimilation and carboxylation rather than direct evidence of increased functional activity. Metabolomic analysis further revealed altered abundances of metabolites associated with central carbon metabolic pathways, particularly glycolysis and the pentose phosphate pathway. This method provides a potential pathway for the value added utilization of by products in the sugar industry by converting waste molasses into MFA type soil amendments. This provides a new organic improvement strategy for improving soil quality, resource recovery, and reducing partial fertilization.

## Data Availability

The original contributions presented in the study are included in the article/Supplementary material, further inquiries can be directed to the corresponding authors.

## Footnotes

1
https://www.genome.jp/kegg/pathway.html

2
https://hmdb.ca/metabolites

3
http://www.lipidmaps.org/
