# Supplementary material for: One-step microbial conversion of waste molasses into bioactive fulvic-like acid fertilizer for sustainable soil quality improvement and agricultural waste valorization
Source: Front Microbiol. 2026 Oct 26;17:1998010. doi: 10.3389/fmicb.2026.1998010 (PMC13745690; doi:10.3389/fmicb.2026.1998010)
Supplement: Supplementary file 1 [file Table_1.DOCX]

S1. Composition of waste molasses components.

Sucrose, glucose, and fructose were determined by high performance liquid chromatography (HPLC). Use carbohydrate column for sugar analysis. Acetonitrile is used as the mobile phase, with a column temperature of 30-40 ℃ and a flow rate of approximately 1.0 mL/min.

K, Ca, Mg, and P were measured using inductively coupled plasma emission spectroscopy (ICP-OES).

The total amount of precipitable colloidal substances is determined using the ethanol precipitation weight method. Take 10 g of molasses, dilute with water and centrifuge, and take the supernatant. Add about 30 ml of anhydrous ethanol, let it stand overnight at 4 ℃, and then dry it to constant weight.

| Material composition | Content (%) |
| --- | --- |
| Sucrose | 30.06 |
| Glucose | 7.42 |
| Fructose | 10.77 |
| Potassium (K) | 3.26 |
| Calcium (Ca) | 0.94 |
| Magnesium (Mg) | 0.37 |
| Phosphorus (P) | 0.18 |
| Water | 24.1 |
| Soluble colloid | 4.56 |

S2. 0.2% MFA field application rate calculation

$$\text{M}_{\text{soil}}\text{=}\text{A}\text{×}\text{D}\text{×}\text{ρ}$$

$$\text{M}_{\text{MFA}}\text{=}\text{M}_{\text{soil}}\text{×0.002}$$

Among them, A is the area, D is the depth of soil mixing, and ρ is the soil bulk density.

Soil bulk density is measured using the ring knife method to determine the dry soil mass corresponding to a unit soil volume. The measured soil bulk density is 1.30 g/cm^3^.

$$\text{ρ}\text{=}\frac{\text{M}}{\text{V}}$$

The depth of the plow layer is 20 cm, and the amount of MFA applied is 5200 kg/ha. The MFA application rate for each 20 m^2^ plot is 10.4 kg.

S3. MFA quantitative analysis

Sample preparation: Take 10.0ml of fermentation broth, centrifuge to remove bacterial cells and suspended solids; Take the supernatant and adjust it to alkaline with NaOH to fully dissolve the humus components; Subsequently, adjust the pH to 2.0 with HCl, let it stand, and centrifuge to precipitate the acid insoluble humic acid; Collect the supernatant that remains dissolved and measure it at a wavelength of 465 nm. Finally, liquid MFA is obtained in solid form through freeze-drying method. And applied in solid form to the field.

MFA production calculation: the proportion of the mass of MFA generated to the mass of waste molasses added.

$$\text{MFA }\text{yield}\text{ (\%) = }\frac{\text{m}_{\text{MFA}}}{\text{m}_{\text{WM}}}\text{×100\%}$$

$$\text{m}_{\text{MFA}}\text{=}\text{C}_{\text{MFA}}\text{×V×DF}$$

Among them, C_MFA_ is the concentration of MFA, V is the extraction volume, and DF is the dilution factor.

Because no commercial certified reference material was available for molasses-derived fulvic-like acid, an in house freeze-dried MFA fraction isolated using the same acid base fractionation procedure was used as the working external calibration material.

Verification using relative standard deviation.

$$\text{RSD=}\frac{\text{SD}}{\overline{\text{π}}}\text{×100\%}$$

The RSD between technical replicate measurements ranged from 1.82% to 3.47%, whereas the RSD among the three independent biological replicates ranged from 4.15% to 6.23%.

Because the acid soluble supernatant may also contain residual inorganic salts, sugars, low molecular weight organic acids, and other soluble compounds, the recovered dry mass was interpreted as an apparent MFA fraction rather than chemically pure MFA.

S4. Metagenomic approach

Total genomic DNA was extracted from soil samples and subjected to shotgun metagenomic sequencing by Novogene Biotechnology Co., Ltd. (Beijing, China). Qualified DNA was randomly fragmented to approximately 350 bp using a Covaris ultrasonicator, followed by end repair, A-tailing, adapter ligation, purification, and PCR amplification. Library integrity and insert-size distribution were evaluated using AATI, and effective library concentrations were determined by qPCR. Qualified libraries were pooled according to their effective concentrations and target sequencing depth and subjected to paired-end 150 bp sequencing (PE150).

Raw sequencing reads were quality-filtered using fastp v0.23.1. Paired reads were discarded when adapter contamination was detected, when low quality bases (Q ≤ 5) accounted for more than 50% of either read, or when ambiguous bases exceeded 10% of the read length. Potential host derived reads were removed by alignment using Bowtie2 v2.5.4, and the resulting high quality clean reads were retained for subsequent analysis.

Clean reads were assembled using MEGAHIT v1.2.9 with the meta-large preset. Scaftigs shorter than 500 bp were removed before gene prediction. Open reading frames (ORFs) were predicted from scaftigs ≥ 500 bp using MetaGeneMark (GeneMark.hmm v2.1), and predicted sequences shorter than 100 nt were discarded.

Predicted ORFs were clustered using CD-HIT v4.5.8 at 95% sequence identity and 90% alignment coverage to construct a non-redundant initial gene catalogue. Clean reads from each sample were mapped back to the gene catalogue using Bowtie2 v2.5.4. Genes supported by ≤ 2 mapped reads across the samples were removed, and the remaining genes constituted the final non-redundant gene catalogue (unigenes). Gene abundance was estimated from the number of reads mapped to each unigene and gene length using a TPM-like normalization procedure.

For taxonomic annotation, unigenes were aligned against the Micro_NR database (version 2024.03) using DIAMOND v2.1.9 with an E-value threshold of 1 × 10^-5^. Taxonomic assignments were determined using the lowest common ancestor (LCA) algorithm. Functional annotation was performed using DIAMOND v2.1.9 against the KEGG (version 2024.03), eggNOG (version 5.0), and CAZy (version 2024.03) databases. For KEGG, eggNOG, and CAZy annotation, alignments were filtered using an E-value threshold of 1 × 10^-5^ and a minimum sequence identity of 30%, and the highest-scoring hit (one HSP > 60 bits) was retained for downstream functional analysis.

Differential taxonomic and functional abundances among treatments were analyzed using MetaGenomeSeq. P values were adjusted for multiple testing using the Benjamini-Hochberg false discovery rate procedure, and q < 0.05 was considered statistically significant. Principal-component analysis and abundance based clustering were additionally performed using the normalized abundance matrices.

The abundances of rbcL, rbcS, accB, accC, and pccA were extracted from the KEGG-annotated metagenomic gene catalogue and compared among treatments. Because shotgun metagenomic sequencing quantifies DNA level gene abundance rather than transcriptional activity, differences in these genes were interpreted as changes in gene abundance and genetic functional potential rather than transcriptional upregulation or direct evidence of increased in situ carbon fixation activity.

Metagenomic abundance normalization and differential analysis. Three independent composite soil samples from three field plots were analyzed for each treatment (n = 3 biological replicates). Clean reads from each sample were mapped to the non-redundant gene catalogue using Bowtie2. Gene abundance was normalized according to mapped read counts and gene length using a TPM-like normalization procedure. Taxonomic and functional abundance analyses were conducted using the normalized abundance matrices. Differential taxonomic and functional features among treatments were identified using MetaGenomeSeq. P values were adjusted for multiple comparisons using the Benjamini-Hochberg false discovery rate (FDR) procedure, and features with q < 0.05 were considered statistically significant.

Untargeted soil metabolomics

Metabolite extraction. Approximately 1.0 g of each soil sample was transferred into a centrifuge tube and extracted with 1.0 mL of 80% (v/v) methanol in water. Samples were vortexed, incubated on ice for 5 min, and centrifuged at 15,000 × g for 15 min at 4 °C. The supernatant was collected and centrifuged again at 15,000 × g for 20 min at 4 °C. The resulting supernatant was freeze-dried and subsequently reconstituted in 10% methanol prior to LC-MS analysis. Pooled QC samples were prepared by mixing equal aliquots of all experimental samples. Blank samples were prepared using 53% methanol in place of the biological sample and were processed in parallel with the experimental samples. Experimental_flow_LC_CH-V1

LC-MS/MS analysis. Metabolomic analysis was performed using a Vanquish UHPLC system coupled to a Q Exactive HF/Q Exactive HF-X high-resolution mass spectrometer (Thermo Fisher Scientific). A Hypersil Gold C18 column (100 × 2.1 mm, 1.9 μm) was used at 40 °C with a flow rate of 0.2 mL min^-1^. The mobile phases consisted of 0.1% formic acid in water (A) and methanol (B). The gradient was 2% B at 0-1.5 min, 85% B at 3 min, 100% B at 10 min, followed by re-equilibration to 2% B. Experimental_flow_LC_CH-V1

The mass spectrometer was operated in both positive- and negative-ion modes over an m/z range of 100-1500. The spray voltage was 3.5 kV, sheath gas flow rate was 35 psi, auxiliary gas flow rate was 10 L min⁻¹, capillary temperature was 320 °C, S-lens RF level was 60, and auxiliary gas heater temperature was 350 °C. MS/MS spectra were acquired using data-dependent acquisition. Experimental_flow_LC_CH-V1

Data preprocessing and normalization. Raw LC-MS data were converted to mzXML format using ProteoWizard and processed using XCMS for peak detection, peak quantification, alignment, and retention-time correction. Metabolites were annotated by matching accurate mass with a mass tolerance of 10 ppm, adduct information, and high-quality MS/MS spectral libraries. Blank samples were used to remove background ions. Relative peak areas were normalized according to total metabolite signal intensity, using the QC1 sample as the reference. Compounds with a CV >30% among QC samples were removed before downstream analysis. Experimental_flow_LC_CH-V1

Metabolite identification confidence. Metabolite identification was classified according to the matching evidence: Level 1, matching of MS1, MS2 and retention time; Level 2, matching of MS1 and MS2; and Level 3, matching of MS1 only. report

Statistical analysis. PCA and PLS-DA were performed using metaX. VIP values were obtained from the PLS-DA model, while univariate significance was evaluated using Student’s t-test. The resulting P values were adjusted for multiple testing using the Benjamini–Hochberg false discovery rate procedure. Differential metabolites were defined as those satisfying VIP >1.0, FC >1.2 or FC <0.833, and FDR-adjusted q <0.05.

S5. Box-Behnken design matrix

| Factor | Symbol | -1 | 0 | 1 |
| --- | --- | --- | --- | --- |
| Temperature (°C) | A | 25 | 35 | 45 |
| Time (h) | B | 24 | 48 | 72 |
| Bacillus subtilis (%) | C | 1 | 5 | 9 |

Response surface experimental design.

| Number | Temperature (℃) | Time (h) | Bacillus subtilis (%) | FA rate (%) |
| --- | --- | --- | --- | --- |
| 1 | 25 | 24 | 5 | 8.13 |
| 2 | 45 | 24 | 5 | 5.05 |
| 3 | 25 | 72 | 5 | 10.66 |
| 4 | 45 | 72 | 5 | 16.84 |
| 5 | 25 | 48 | 1 | 5.56 |
| 6 | 45 | 48 | 1 | 4.55 |
| 7 | 25 | 48 | 9 | 14.68 |
| 8 | 45 | 48 | 9 | 13.59 |
| 9 | 35 | 24 | 1 | 3.99 |
| 10 | 35 | 72 | 1 | 12.47 |
| 11 | 35 | 24 | 9 | 11.53 |
| 12 | 35 | 72 | 9 | 17.42 |
| 13 | 35 | 48 | 5 | 33.54 |
| 14 | 35 | 48 | 5 | 31.77 |
| 15 | 35 | 48 | 5 | 32.46 |
| 16 | 35 | 48 | 5 | 30.69 |
| 17 | 35 | 48 | 5 | 32.55 |

Regression equation:

Y=32.202+0.125A+3.59B+3.83C+2.31AB-0.02AC-0.6475BC-11.89A^2^-10.14B^2^-10.71C^2^

Statistical parameter

| Model F-value | 126.8 |
| --- | --- |
| Model p-value | ＜0.0001 |
| R^2^ | 0.9939 |
| Adjusted R^2^ | 0.9861 |
| Predicted R^2^ | 0.9355 |
| Lack-of-fit F-value | 2.22 |
| Lack-of-fit p-value | 0.2286 |

ANOVA

| Source | Sum of squares | df | Mean square | F-value | p-value |
| --- | --- | --- | --- | --- | --- |
| Model | 1931.84 | 9 | 214.65 | 126.80 | <0.0001 |
| Residual | 11.85 | 7 | 1.69 | — | — |
| Lack of fit | 7.40 | 3 | 2.47 | 2.22 | 0.2286 |
| Pure error | 4.45 | 4 | 1.11 | — | — |
| Total | 1943.69 | 16 | — | — | — |

The optimal prediction conditions of the model are 35.22 ℃, 52.17 h, and 5.69%. According to the regression equation, the predicted conversion rate of MFA is 32.85%. We conducted three validation experiments under conditions of 35 ℃, 52 h, and 5.7%. The final average conversion rate of MFA was 31.91%. The average MFA conversion rate of 32.2% obtained under the conditions of 35 ℃, 48 h, and 5% at the center point is slightly smaller and there is no significant difference. In order to be more in line with reality and save resources, fermentation conditions of 35 ℃, 48 h, and 5% are adopted.

S6. Analysis of GC-MS characteristic substance results.

| GC-MS Name | 0 h | 24 h | 48 h |
| --- | --- | --- | --- |
| Butanoic acid, 2-methyl-, ethyl ester | 8.49% | 5.37% | 1.1% |
| Butanoic acid, 2-methyl-, propyl ester | 6.16% | 3.11% | 1.84% |
| Decanoic acid, ethyl ester | 2.58% | 4.26% | 0.53% |
| 1-Dodecanol | 6.54% | 8.22% | 3.69% |
| 1-Tetradecanol | 9.11% | 9.41% | 1.22% |
| Phenol, 2-methoxy | 10.02% | 2.06% | 0.11% |
| Phenol, 4-ethyl | 9.13% | 1.34% | 0.09% |
| Trans-Isoeugenol | 0.36% | 0.22% | 0.31% |
| Tetradecane | 2.66% | 5.38% | 11.93% |
| Heptadecane | 2.57% | 4.32% | 0.49% |
| Hexanedioic acid, bis (2-methylpropyl) ester | 1.07% | 6.54% | 0.86% |
| 4-Hydroxy-2-methylacetophenone | 8.55% | 41.57% | 4.37% |
| Nonanal | 2.77% | 10.09% | 0.26% |
| Dodecanal | 3.2% | 5.31% | 0.17% |
| Phenol, 2,6-dimethoxy | 15.48% | 8.34% | 1.05% |

24 h

0 h

48 h

S7. Analysis of LC-MS characteristic substance results.

| LC-MS Name | 0 h | 24 h | 48 h |
| --- | --- | --- | --- |
| Sinapinic acid | 18.36% | 7.85% | 1.25% |
| Syringic acid | 12.77% | 8.01% | 0.38% |
| 2,6-Dimethoxyphenol | 12.94% | 7.44% | 1.06% |
| Ethyl Vanillate | 13.24% | 6.97% | 3.05% |
| L-α-Palmitin | 6.52% | 3.07% | 2.04% |
| L-Phenylalanine | 6.39% | 4.53% | 3.07% |
| L-Isoleucine | 2.66% | 1.27% | 0.47% |
| L-Tyrosine | 4.24% | 2.31% | 1.02% |
| 4-oxododecanedioic acid | 1.59% | 28.32% | 10.26% |
| 3-hydroxy-2-octylpentanedioic acid | 3.23% | 25.84% | 7.35% |
| 3-(4-hydroxy-3-methoxyphenyl) Propanoic acid | 0.12% | 8.61% | 2.73% |
| Isovanillic acid | 0.56% | 3.39% | 4.62% |

24 h

0 h

48 h

S8. Analysis of C, H, O, and N element content in MFA

| Element | Content (%) |
| --- | --- |
| C | 32.61 |
| H | 4.40 |
| O | 30.58 |
| N | 1.21 |

S9. Plant growth status.

| Treatment | plant height (cm) | stem diameter (cm) | Leaf Length (cm) | dry weight  (g) |
| --- | --- | --- | --- | --- |
| CK | 142.34±3.42 | 1.16±0.05 | 50.93±2.37 | 110.52±4.14 |
| CN | 248.51±4.01 | 2.63±0.11 | 88.36±4.52 | 284.26±4.88 |
| CF | 230.06±2.15 | 2.41±0.08 | 80.18±4.06 | 237.96±4.35 |
